# The Potential of Liming to Improve Drought Tolerance of Norway Spruce [*Picea abies* (L.) Karst.]

**DOI:** 10.3389/fpls.2019.00382

**Published:** 2019-03-29

**Authors:** Martin Kohler, Jörg Kunz, Johannes Herrmann, Peter Hartmann, Lelde Jansone, Heike Puhlmann, Klaus von Wilpert, Jürgen Bauhus

**Affiliations:** ^1^Faculty of Environment and Natural Resources, Institute of Forest Sciences, Chair of Silviculture, University of Freiburg, Freiburg, Germany; ^2^Department of Soils and Environment, The Forest Research Institute Baden-Wuerttemberg, Freiburg, Germany

**Keywords:** Norway spruce, liming, drought tolerance, resistance, resilience

## Abstract

In response to a wide-spread decline in forest vitality associated with acid rain in the 1980s, liming of soils has been implemented in many federal states in Germany to buffer further acid deposition and improve availability of nutrients such as calcium and magnesium. As a consequence, it may also increase vitality and depth of fine-root systems and hence improve the drought tolerance of species such as Norway spruce [*Picea abies* (L.) Karst.], which occurs mostly on acidic forest soils. However, the influence of repeated liming on drought tolerance of trees has never been studied. Here we compared the resistance, recovery and resilience of radial growth in *P. abies* in relation to drought in limed and control stands and assessed how the dosage and interval between lime application and drought year influences the radial growth response of *P. abies*. We analyzed radial growth in 198 *P. abies* trees of six experimental sites in south–west Germany. An analysis of the radial increment over the last 30 years allowed the analysis of drought events shortly after the first liming (short-term effect) as well as posterior drought events (mid- to long-term effects). Generalized linear models were developed to assess the influence of drought intensity, site and period since first liming on the drought tolerance of Norway spruce. Regardless of drought intensity, there was no general increase in drought resistance of Norway spruce in response to liming. However, drought resistance of radial growth improved on a loamy site that was additionally treated with wood ash 30 years after the first lime application. Furthermore, recovery and resilience of radial growth after severe drought events were generally better in spruce trees of limed treatments. This indicates a shorter stress period in spruce trees growing on limed soil, which may reduce their susceptibility to secondary, drought-related pests and pathogens.

## Introduction

The adaptation of forests to climate change is currently a major challenge of silviculture. In Central Europe, this applies in particular to forests dominated by Norway spruce [*Picea abies* (L.) Karst.] which has been cultivated on many sites, where it will no longer be suitable or associated with high production risks under future climate conditions (Hanewinkel et al., 2013). On the other hand, Norway spruce is still the economically most important tree species in Germany. According to the results of the last German Forest Inventory from 2012 (BWI III), Norway spruce has a share of 25% of the total forest area and will dominate the German raw timber supply until the middle of this century (BMELV, 2018).

To improve the tolerance of Norway spruce stands against climate change impacts, different silvicultural strategies can be pursued. In the long-term, more drought tolerant tree species may be admixed or used to replace Norway spruce (Vitali et al., 2017). In the short-term, stability and vitality of existing trees and stands may be increased through silvicultural manipulations such as thinning that provide more growing space to trees (Kohler et al., 2010; Sohn et al., 2012).

Likewise, liming is assumed to promote the vitality of forest stands on acidic soils. In many federal states of Germany, liming has been practiced since the early 1980s in response to a wide-spread decline in forest vitality associated with acid rain. For this purpose, mostly magnesium-containing limestone powder (dolomite) has been applied in dosages of ca. 3 t ha^−1^ every 10 years. The main goal of these lime applications has been to increase the buffering of the acidity input from atmospheric deposition to counteract the man-made soil acidification and loss of soil quality (Ulrich, 1986; Huettl, 1989; Wilpert et al., 2013). Furthermore, lime application may increase the availability of Ca, Mg, and possibly other nutrients to trees, stimulate decomposition of forest floor material and its incorporation in the mineral soil, and increase fine-root vitality and proliferation in deeper soil layers (Schäffer, 2006; Wilpert et al., 2013).

Based on these effects of liming, it has been proposed that liming should be continued to be applied on acidic soils to increase the drought tolerance of forests (Landesanstalt für Umwelt Messungen und Naturschutz Baden-Württemberg [LUBW], 2013). However, there has been no study that has explicitly analyzed the effects of liming on the drought tolerance of forests. Mostly, existing liming experiments addressed short to medium-term effects on soil properties and forest nutrition (Ulrich, 1986; Huettl, 1989; Kreutzer, 1995; Ponette et al., 1997; Schaaf and Hüttl, 2006). Few studies considered long-term effects on tree growth. Following a once-only lime application of 2 t ha^−1^, the growth of Norway spruce in the Northern Black Forest increased for more than 25 years (Spiecker, 1991). In that study, also interannual weather effects on the growth response after liming have been described, however, resistance and resilience of tree growth in relation to particular drought events were not evaluated quantitatively. In Norway spruce stands limed once with 2.5 t ha^−1^, a two-phase growth response with a strong increase (plus 54%) in the first 8 years after liming, and a subsequent decrease to a growth level which remained considerably higher than in the control stand (plus 20%) was observed (van der Perre et al., 2012). Particularly in the second phase (>8 years after liming), the interannual variability of the “growth gain associated to liming” (van der Perre et al., 2012) increased significantly and could be partially explained by interannual variation in weather conditions. This increase in “weather-sensitivity” of radial growth after liming was explained through the Norway spruce-specific, shallow root system as well as the limited “depth effect” of only one liming measure (van der Perre et al., 2012). Corresponding to these results, there are several studies that found even more shallow fine-root systems of Norway spruce after liming (Kreutzer, 1995; Helmisaari and Hallbäcken, 1999). This would mean that liming may rather increase the risk for drought stress. In contrast, Schäffer (2006) found for Norway spruce a deeper and more homogeneous root penetration as well as higher fine root densities in the mineral soil, even more than 50 years after lime application. However, the lime dosage in that experiment was extremely high (10 t ha^−1^).

To what extent repeated applications of lime actually improve drought tolerance of single trees or forest stands remains unclear. There have been no studies on the growth response during (resistance) and following drought years (recovery and resilience) in relation to the dosage and number of lime applications as well as the interval between lime application and the drought event. Therefore, the hypotheses addressed in this study were:

(1) Resistance, recovery and resilience of radial growth in *P. abies* in relation to drought are higher in limed than in control stands.
(2) Effects of liming on radial growth resistance, recovery and resilience of *P. abies* are influenced by site, the number of lime applications, as well as the period between lime application and drought event.

## Materials and Methods

### Study Sites

The study sites are part of a long-term liming experiment in the German federal state of Baden-Württemberg, which was initiated in the beginning of the 1980s. We selected six sites (Table 1) which are located in different climatic regions and representing typical forest soils in those regions. The sites are distributed over an area extending 270 km in north–south (Heidelberg to Bad Waldsee) and about 120 km in west-east (Herzogenweiler to Hospital) directions. They represent a broad and typical variety of Norway spruce stands in South–West Germany. Sites are characterized by a homogeneous topography to exclude expositional effects on tree growth.

**Table 1:** Summary information on main characteristics of the experimental sites.

	Bad Waldsee	Heidelberg	Herzogenweiler	Horb	Hospital	Weithard
Latitude [°]	47°50′	49°30′	48°01′	48°28′	48°07′	47°58′
Longitude [°]	9°41′	8°47′	8°20′	8°32′	9°41′	9°17′
Mean annual temperature [°C] 1971–2013	8.7	9.4	6.8	8.4	8.2	7.9
Annual precipitation [mm] 1971–2013	970	1130	1190	1160	840	830
Altitude [m asl]	580	490	950	630	650	630
Main tree species	*P. abies*	*P. abies*	*P. abies*	*P. abies*	*P. abies*	*P. abies*
Mixed tree species	*F. sylv., L. dec.*		*A. alba*	*A. alba*	*F. sylv., L. dec.*	
Soil types	Cambisols	Podsols	Cambisols (stagnosols, luvisols)	Cambisols (stagnosols, luvisols)	Stagnosols (luvisols)	Stagnosols (cambiosols, luvisols)
Parent material	Glacial till (Würm stage)	Sand stone (middle lower Trias)	Sand stone (upper lower Trias)	Sand stone (upper lower Trias)	Glacial till (Riss stage)	Glacial till (Würm stage)
Soil texture class	Loam	Sandy loam	Loam	Clay loam	Loam – silty loam	Loam
Humus types	Mull to moder mull	Mull	Mull to moder mull	Mull	Mull to mor moder	Mull to mor moder

*Mixed tree species are abbreviated as F. sylv. (Fagus sylvatica L.), L. dec. (Larix decidua MILL.), and A. alba (Abies alba MILL.). Meteorological data derived from Dietrich et al. (2018).*

All sites received the first application of dolomite of 3 t ha^−1^ in 1985 and contained an untreated control. In 2003, all sites were limed again with 6 t ha^−1^ of pure dolomite. The higher dosage was chosen to trigger more pronounced effects of liming on soils and forest stands than could be observed until then. At sites Heidelberg, Horb and Weithard, the third lime application 2015 was done with a 3.85 t ha^−1^ dolomite – wood ash mixture in a ratio of 3:1 whereas at sites Bad Waldsee, Herzogenweiler and Hospital common dolomite (3.85 t ha^−1^) was applied also in 2015. The wood ash was taken only from the combustion of untreated woods and subject to thorough quality control (Wilpert et al., 2016).

Soils were sampled at five plots per treatment down to a depth of 60 cm in May 2015 using root corers with a diameter of 8 cm. In the field, fine-root tips were counted at the core surfaces in 5 cm depth increments. Soil chemical variables were determined according to the protocol of the Gutachterausschuss forstliche Analytik (2009). The pH was measured in 1 M KCl and cation exchange capacity in 1M NH_4_Cl-solution. Base saturation was calculated as the percentage ratio of the sum of the ion equivalents of Ca, K, Mg, and Na to the cation exchange capacity. All soil chemical properties of the limed sites represent soil properties before liming in 2015. As indicated by pH values between 3 and 4 (Table 2), soils at all study sites were very acidic. Previous liming had either no (soil depth 21–60 cm) or only a moderate effect (soil depth 0–20 cm) on pH. In contrast, liming had considerably increased base saturation, especially in the top mineral soil at 0–20 cm depth meaning that the risk for deficiencies in basic nutrients – which may occur at these low soil pH levels – had been reduced after liming.

**Table 2:** Mean pH values and base saturation (BS) in upper (0–20 cm) and lower (21–60 cm) mineral soil layers at study sites (measured before liming campaign 2015).

Site	Bad Waldsee		Heidelberg		Herzogenweiler		Horb		Hopsital		Weithard	
Treatment	Liming	Control	Liming	Control	Liming	Control	Liming	Control	Liming	Control	Liming	Control
pH (KCl) 0–20 cm	3,8	3,4	3,8	3,0	3,7	3,4	3,7	3,4	3,6	3,4	3,7	3,5
pH (KCl) 21–60 cm	4,1	3,9	4,0	4,1	3,9	3,9	3,8	3,7	3,7	3,7	3,8	4,0
BS [%] 0–20 cm	33,0	9,0	82,0	8,0	30,0	4,0	41,0	20,0	29,0	8,0	37,0	8,0
BS [%] 21–60 cm	10,0	6,0	26,0	5,0	9,0	4,0	8,0	13,0	14,0	18,0	14,0	7,0

**FIGURE 1:**
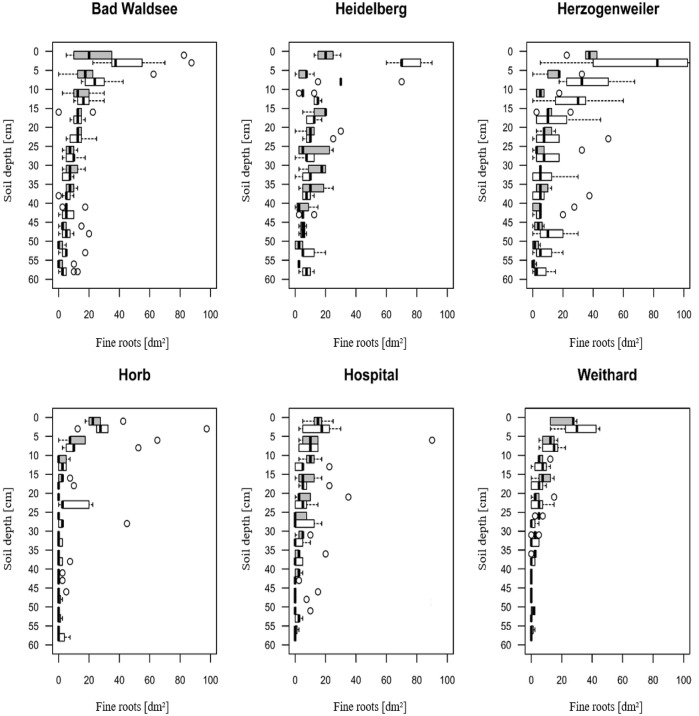
Fine-root densities (tips per dm^2^ soil) in 0 to 60 cm soil depth at study sites (measured before liming campaign 2015). Gray box plots indicate the control group; white box plots denote the liming treatment. Circles indicate outliers.

Also, liming lead to higher numbers of fine roots, mainly in the topsoil. This effect was strongest at the Herzogenweiler site and weakest at Hospital (Figure 1).

### Sampling of Tree Cores and Measurement and Processing of Tree-Ring Series

Trees selected for coring showed no visible signs of pathogens, needle abscission, dieback or other damages. Additionally, diameter at breast height (dbh, 1.3 m), and height were measured for all sample trees. The ratio of height over dbh was used as an indicator for single tree stability and competition pressure (Albrecht et al., 2012). Using height and age data of trees, site indices according to yield tables of Schober (1995) (mean annual increment for 100 years = MAI_100_) were derived. Except for the site Horb, no distinct differences in tree and stand variables (e.g., height, diameter in breast height, stand age) could be observed between the two treatments. At Horb, differences in tree variables between the two treatments could be attributed to the younger age of the control (by 35 years) when compared to the limed stand (Table 3). Overall, growth properties observed in trees from both treatments have been within the normal range being typical for Norway spruce stands in Baden-Württemberg (BWI III).

**Table 3:** Mean tree height, diameter at breast height (dbh), height to diameter ratio (h/d), stand age and site index of sampled *P. abies* trees.

Site	Bad Waldsee		Heidelberg		Herzogenweiler		Horb		Hospital		Weithard	
Treatment	Liming	Control	Liming	Control	Liming	Control	Liming	Control	Liming	Control	Liming	Control
Tree height [m]	33.3	32.7	33.5	34.2	31.2	31.9	33.6	30.1	32.8	32.8	35.5	34.9
dbh [cm]	48.4	47.0	48.0	47.1	43.2	46.8	45.5	34.4	47.2	46.5	55.2	50.1
h/d ratio	69.7	70.3	71.1	73.5	72.3	68.8	74.3	88.2	70.7	71.0	65.3	69.9
Stand age [yr]	70	70	75	75	85	95	95	60	80	65	90	75
Site index [m^3^yr^−1^]	15.3	15.0	14.7	15.0	11.3	10.8	11.8	14.7	13.4	15.8	14.0	15.7
*N* sample trees	16	16	18	18	16	16	17	17	16	16	16	16

Increment cores were taken in the summers of 2016 (Heidelberg, Horb, Weithard) and 2017 (Bad Waldsee, Herzogenweiler, Hospital) from 198 *P. abies* trees of (co-) dominant canopy status. Sample trees were evenly distributed among both treatments at each site. From each tree, two increment cores were taken at breast height in northern and western cardinal directions. Cores were dried and subsequently sanded to obtain clean surfaces for the analysis of radial increments. To determine the radial increment, tree-ring widths were measured to the nearest 0.01mm using a Lintab digital positioning table (Rinntech, Heidelberg, Germany) in conjunction with the time series analysis program TSAP (Rinntech, Heidelberg, Germany). Individual tree-ring series were cross-dated visually using the TSAP software, also an interval sign test (Gleichläufigkeit) was applied (Schweingruber, 1983). Ring series that could not be synchronized were removed from further analyses. Tree-ring series from the two cores of one tree were averaged and subsequently, series were transformed to basal area increment (BAI) which is less biased by stem geometry or age trends (West, 1980) and hence expected to provide better dendrochronological estimates (LeBlanc, 1990). Afterwards, BAI series were detrended by applying a smoothing spline function with a 50% frequency cutoff of 2/3 of the series length to remove long-term trends owing to age, size and stand dynamics (Fritts, 1976). Calculation of basal area increment, detrending and the quality control of the tree-ring data were conducted using the software *R* 3.4.4 ^1^ and the packages *dplR* (Bunn, 2008), and *detrendeR* (Campelo et al., 2012).

Dendrostatistical indices were used to assess the accuracy and quality of tree-ring data. We calculated mean sensitivity (ms), first-order autocorrelation (ar1), cross-correlation between single series (rbar), and the expressed population signal (EPS) (Fritts, 1976; Wigley et al., 1984). The computed dendrochronological indices (Table 4) indicated an overall good accuracy of the used tree-ring data. Mean sensitivity ranged between 0.21 and 0.28, which is common for trees of temperate forests (Fritts and Shatz, 1975). Also, the values for ar1 and rbar were within a favorable range (Lebourgeois et al., 2005). Although the validity of the expressed population signal itself is highly discussed (Buras, 2017), the EPS values for all sites and treatments were clearly above 0.85, which is commonly used as an accuracy threshold for studies of tree populations (Wigley et al., 1984).

**Table 4:** Dendrochronological indices (mean sensitivity (ms), first-order autocorrelation (ar1), cross-correlation between single series (rbar), and the expressed population signal (EPS) for *P. abies* trees from all study sites and treatments.

Site	Bad Waldsee		Heidelberg		Herzogenweiler		Horb		Hospital		Weithard	
Treatment	Liming	Control	Liming	Control	Liming	Control	Liming	Control	Liming	Control	Liming	Control
ms	0.263	0.260	0.267	0.287	0.222	0.211	0.260	0.257	0.268	0.255	0.281	0.263
ar1	0.774	0.746	0.812	0.746	0.858	0.859	0.876	0.892	0.732	0.794	0.876	0.863
rbar	0.533	0.519	0.754	0.673	0.795	0.670	0.830	0.835	0.508	0.623	0.784	0.706
EPS	0.939	0.928	0.975	0.957	0.983	0.966	0.981	0.981	0.938	0.958	0.975	0.959

### Identification of Drought Years

To identify and classify retrospectively drought events that occurred at the study sites, the non-dimensional, multi-scalar Standardized Precipitation Index (SPI) was used (McKee et al., 1993). To calculate the index, site specific data of monthly precipitation sum provided by the Forest Research Institute of Baden-Wuerttemberg (FVA) were used. Meteorological data were derived from regionalized data according to Dietrich et al. (2018). Data gaps were filled with time series of monthly precipitation sums from nearby meteorological stations of the German National Meteorological Service (DWD). All meteorological stations were located less than 15 km from the study sites and at a comparable altitude (Kunz et al., 2018). In case that more than one station was available, the data of the additional stations were used to control quality of the meteorological time series (Mayer et al., 2005). Following the recommendations by Hayes et al. (1999), SPI-3 (June to August precipitation of the current year) time series were calculated from 1961 to 2016. The calculation of the SPI was conducted using the software *R* 3.4.4 and the package *spi* (Neves, 2012).

For an accurate interpretation of the index, objective drought thresholds have to be defined (Quiring, 2009). According to McKee et al. (1993), SPI values up to −1 define normal conditions, SPI values between −1 and −1.49 represent ‘moderate drought’ conditions, SPI values between −1.5 and −1.99 indicate ‘severe drought’, and SPI values ≤-2 indicate ‘extreme drought’. In our study, years in which at least moderately dry periods were present (SPI values below −1), were selected as ‘drought years’ and further studied. The most common and distinct drought years were 1962, 1983, and 2003, which were detectable at all six study sites (Table 5). The years 1964, 1998, and 2015 were detectable as drought years at four study sites. The most extreme single drought event was detected 1983 in Weithard, where a SPI value of −3.48 was reached. After the start of the liming experiment in 1985, only two extreme drought events have been detected with SPI-3 value of −2.35 in Bad Waldsee in year 2003 and SPI-3 value of −2.13 in Horb in year 2015.

**Table 5:** Summary of the SPI-3 values calculated for the months June to August of the current year.

Bad Waldsee		Heidelberg		Herzogenweiler		Horb		Hospital		Weithard	
Year	SPI-3	Year	SPI-3	Year	SPI-3	Year	SPI-3	Year	SPI-3	Year	SPI-3
**2003**	−2.35	**1983**	−2.85	**1983**	−2.30	**1983**	−2.74	**1983**	−2.71	**1983**	−3.48
**1983**	−2.27	1976	−2.36	1991	−1.73	2015	−2.13	**1962**	−2.45	**2003**	−1.70
1998	−1.92	**2003**	−1.91	1998	−1.73	1981	−1.75	1998	−1.92	1964	−1.68
**1962**	−1.85	1964	−1.66	1985	−1.71	**1962**	−1.65	**2003**	−1.62	2015	−1.37
2015	−1.64	**1962**	−1.58	1988	−1.43	1976	−1.58	1964	−1.57	1981	−1.35
1981	−1.38			1994	−1.22	**2003**	−1.55	2015	−1.21	1984	−1.22
2004	−1.35			**1962**	−1.20	1989	−1.49	2006	−1.06	**1962**	−1.11
2006	−1.18			1984	−1.14	1964	−1.44			1972	−1.02
				1974	−1.12					2013	−1.02
				**2003**	−1.12					1998	−1.01
				2005	−1.01						

*Only years with at least ‘moderate drought’ are shown (SPI-3 values below −1). Drought years are ordered by increasing SPI-3 values. Years in bold characters indicate droughts year that were detectable at all six study sites. SPI-3 values in light gray cells indicate ‘moderate drought years,’ mid gray cells mark ‘severe drought years,’ and dark gray cells represent ‘extreme drought years.’*

### Responses of Radial Growth to Climatic Variations

We conducted retrospective analyses of radial growth before, during, and after drought years. For this purpose, indices for resistance (*Rt*), recovery (*Rc*), and resilience (*Rs*) of radial growth in relation to moderate, severe, and extreme drought years were calculated following the approach of Lloret et al. (2011). In general, resistance, recovery and resilience can be seen as components of a species’ ability to tolerate stress (Pimm, 1984), where tolerance is defined as the potential of a single species or the whole ecosystems to withstand stress and/or swing back into a stable state following stress exposure without changing into a new system (Peterson et al., 1998). Against this background, we calculated these drought tolerance indices as follows:

(1) $$\mathrm{Rt}=\frac{\mathrm{BAI DY}}{\mathrm{BAI PreDY}}$$

(2) $$\mathrm{Rc}=\frac{\mathrm{BAI PostDY}}{\mathrm{BAI DY}}$$

(3) $$\mathrm{Rs}=\frac{\mathrm{BAI PostDY}}{\mathrm{BAI PreDY}}$$

where BAI DY is the detrended annual basal area increment (BAI) during the drought year, BAI PreDY is the BAI during the years before the drought, and BAI PostDY is the BAI during the years after the drought of individual trees (Lloret et al., 2011). Based on Kunz et al. (2018) who found that neither extended, nor shortened reference periods between 3 and 5 years changed results in the calculation of resistance, recovery, and resilience significantly, we used a period of 5 years before and after the drought event as reference periods for the calculation of these indices. No indices of recovery and resilience could be calculated for the drought year 2015 as increment cores were taken in summer 2016 and 2017. The calculation of the drought tolerance indices was conducted using the software *R* 3.4.4 and the package *pointRes* (van der Maaten-Theunissen et al., 2015).

### Statistics

We used generalized linear models (GLMs) to analyze to what extent factorial variables such as “treatment” (limed, control) or “site” and continuous variables such as “stand age at drought event,” “site index,” and “time period between first liming and drought year” (Δt) explained variation in Rt, Rc, and Rs. Originally, we intended also to test the “time period between the last liming and drought year” (Δ*t_last_*) and the “total dosage of lime applied at drought event.” However, as a result of the temporal distribution of the three lime applications and the identified drought years we had finally too few data to establish models including these latter two variables. For non-limed control plots, we also assigned corresponding Δ*t* values so that we could test in joint models whether possible Δ*t* effects could exclusively be attributed to liming or occurred also in non-limed control plots.

We used GLMs as our target variables were mainly not normally distributed and could also not be consistently normalized through common transformation methods. We fitted separate models for each of the three drought intensity types (moderate, severe, and extreme droughts). The model parametrization was done stepwise considering all available continuous and factorial variables. Non-significant variables were excluded from the model if this led to a simpler model with a more favorable AIC (Akaike’s information criterion). We also considered interactions between variables, especially between site and treatment to check for site-specific liming responses. In cases, where we found models with a positive response to liming, we additionally tested the influence of time period between first liming and drought year by adding the variable Δ*t* to the best model of the corresponding target variable.

All data analysis was done with the software R 3.4.4. Tests for normal distribution were conducted using the package “nortest.” Generalized linear models were fitted using the “gls” function from the package “nlme.” Model selection was done using the package “MuMIn” based on AIC. Model visualization was carried out using the package “visreg.”

## Results

### Radial Increment Series of Sample Trees

**FIGURE 2:**
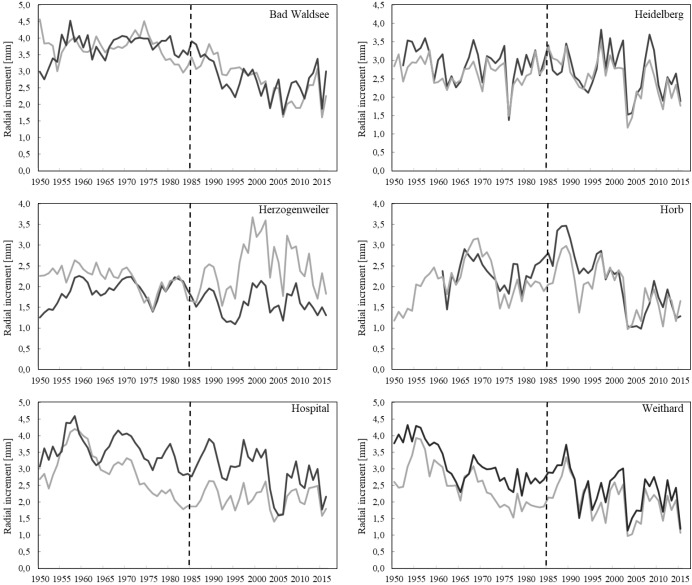
Mean radial increment of *P. abies* trees in limed treatments (gray lines) and controls (black lines) of the different study sites. Lime application started in 1985 (dashed line).

When comparing the mean radial increment series of Norway spruce trees between treatment and control groups, no positive effect of liming on diameter growth was evident except for the Herzogenweiler site (Figure 2). This was in accordance to the positive effects of liming on fine root distribution (Figure 1) which was strongest at Herzogenweiler. At Hospital, radial increments of trees in the control were continuously – before and after the liming experiment had started – higher than in the limed treatment. This may be explained with differences in age-related growth dynamics at this site (Table 3). Similarly, at Weithard, the limed site had initially lower radial increments than the control but within 5 years after the lime application it reached the growth level of the control. This can be interpreted as liming effect. At all study sites, the long-term mean radial increment ranged between 2 and 3 mm year^−1^.

### Drought Resistance of BAI (Rt)

Model results showed that site was the predictor variable that explained most variation in resistance of *P. abies* across all drought intensities (Table 6).

**Table 6:** Model results (glm) for the BAI resistance (Rt) to drought of *P. abies.*

	Predictors for Rt								
Drought type	Treatm. control	Site	mai_100_	Stand age at drought	Elevation	Site × Treatment	AIC	Delta	AIC weight
**Moderate**		**X**					−318	0	0.66
(*N* = 348)	**X (**−**3.3%)**	**X**		x			−316	2	0.30
	x	**X**					−312	6	0.03
				x			−279	39	0.00
	**X (**−**3.5%)**			x			−277	41	0.00
**Severe**		**X**					−213	0	0.97
(*N* = 300)	x	**X**					−206	7	0.03
	x	**X**		x			−195	18	0.00
					**0.0005**		−122	91	0.00
	x				**0.0005**		−116	97	0.00
**Extreme**			−**0.0529**				−46.1	0	0.39
(*N* = 58)		**X**					−45.5	0.6	0.28
	x	**X**				**X**	−44.9	1.2	0.21
	**X (**−**9.1%)**	**X**					−43.3	2.8	0.09
	x		−**0.0502**				−39.9	6.2	0.02

*Only the best fitting five models according to the AIC values are listed for moderate droughts (SPI −1 to −1.49), severe droughts (SPI −1.5 to −1.99) and extreme droughts (SPI ≤-2), respectively. X indicates significant factorial predictors, x indicates non-significant factorial predictors and bold figures provide the slope of significant continuous predictors. For the factor “Treatment” also the relative intercept change from control to limed treatment is listed.*

**FIGURE 3:**
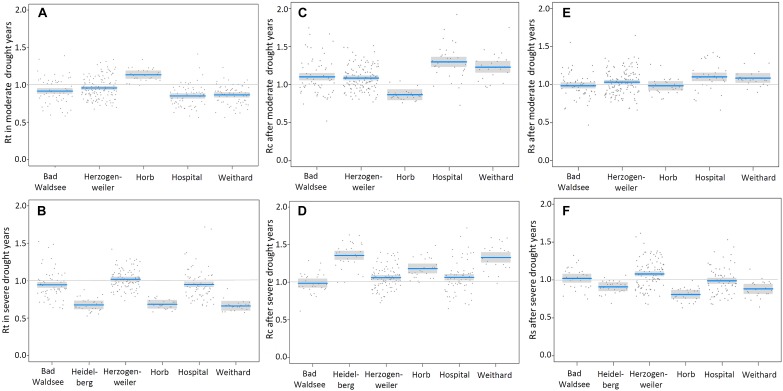
Resistance “Rt” **(A,B)**, recovery “Rc” **(C,D)** and resilience “Rs” **(E,F)** of detrended annual basal area increment in relation to moderate (upper panels) and severe drought years (lower panels) at the different sites (blue lines: modeled values of Rt, Rc and Rs; gray bands: confidence interval for the expected value; dark gray dots: residuals).

In moderate drought years, the BAI in spruce trees at Horb was not reduced and at all other sites only slightly reduced, respectively (Figure 3A). The strongest growth depression of 15% compared to the pre-drought level was detected at Hospital. In the second best model, we also found for the factor treatment a significant effect. However, the effect size was almost negligible as the resistance (Rt) was only 3% better at limed than at control sites (Table 6). We also tested models considering potential interactions between site and treatment, but no site-specific treatment effect could be detected.

In years classified as severe droughts, we found no treatment effect at all but the site-specific resistance to drought (Rt) varied considerably. At Heidelberg, Horb and Weithard, radial growth was reduced to less than 70% of the pre-drought level, whereas only a slight reduction at Bad Waldsee (−6%) and Hospital (−5%) and almost no change of Rt at Herzogenweiler could be observed (Figure 3B).

**FIGURE 4:**
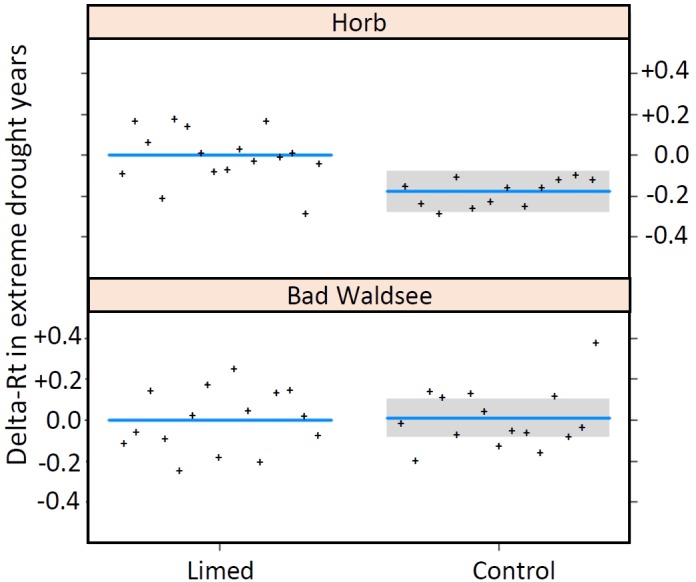
Interaction model (Site × Treatment) for the resistance of radial growth (rt) in extreme drought years expressed as the difference (ΔRt) between control and limed treatment. Extreme drought events (SPI ≤ –2) occurred only in 2015 at Horb and 2003 at Bad Waldsee (plots created by R function “visreg, type = contrast,” therefore confidence bands are not given for the reference “Limed”).

Owing to the low number of extreme drought years in the 30-year period after first lime application in 1985, only the two study sites Bad Waldsee and Horb and the two drought years 2003 and 2015 could be used for model analyses. The three best models with comparable AICs between −46 and −45 (Table 6) identified a better resistance (Rt) at site Horb (+25%), a negative influence of site index on Rt (with a slope of −0.053 per unit site index) and a site-specific treatment effect at site Horb where Rt of limed Norway spruce trees were distinctly better (+20%) compared to control trees while at Bad Waldsee no treatment effect could be detected (Figure 4).

### Growth Recovery After Drought (Rc)

For Rc and likewise for Rs (chap. 3.4), modeling included only moderate and severe droughts as for the second of the two detected extreme drought events (2003 at Bad Waldsee and 2015 at Horb) post drought year tree-ring data were not available (sampling at Horb in summer 2016) and the remaining sample size was not sufficient to establish valid models.

Also for recovery (Rc), site was the most distinct factor to predict radial growth recovery of Norway spruce, regardless of drought intensity. Considering the best models for moderate drought years, no treatment effect on Rc could be detected (Table 7). The recovery of BAI after moderate droughts compensated at least growth depressions during previous drought years at all sites with the exception that at Horb the index of Rc indicated a growth reduction of about 15% in the 5 post drought years (Figure 3C). However, this Rc reduction corresponded exactly to the amount of additional growth in the dry year (Rt), indicating that after the dry year growth rate returned to the “normal growth” before moderate drought years (Figure 3A).

**Table 7:** Model results (glm) for the BAI recovery (Rc) of *P. abies* after drought.

	Predictor for Rc								
Drought type	Treatm. control	Site	mai_100_	Stand age at drought	Elevation	Site × Treatment	AIC	Delta	AIC weight
**Moderate**		**X**					−99	0	0.98
(*N* = 270)	x	**X**					−91	8	0.02
	x	**X**		0.0011			−79	20	0.00
	x	**X**				**X**	−77	22	0.00
			**0.0142**				−45	54	0.00
**Severe**	**X (**−**7.8%)**	**X**					−161	0	0.99
(*N* = 139)		**X**					−152	9	0.01
	**X (**−**8.1%)**	**X**		0.0007			−148	13	0.00
	x	**X**				**X**	−137	24	0.00
	**X (**−**6.4%)**				−**0.0003**		−88	73	0.00

*Only the best fitting five models according to the AIC values are listed for moderate droughts (SPI −1 to −1.49) and severe droughts (SPI −1.5 to −1.99) respectively. X indicates significant factorial predictors, x indicates non-significant factorial predictors and bold figures provide the slope of significant continuous predictors. For the factor “Treatment” also the relative intercept change from control to limed treatment is listed.*

For severe droughts, models showed clearly that site and treatment were the most important factors to predict Rc. Based on the best model (AIC = −161), the growth recovery after severe drought was approximately 8% better in trees from limed sites compared to controls (Table 7). However, Rc varied to a much greater extent between sites (Figure 3D). At Bad Waldsee, Herzogenweiler and Hospital, Rc indices were close to 1.0 which was already observed for Rt, meaning that radial growth was only slightly affected during and after severe droughts at these sites. In contrast, the growth recovery at sites which showed highest growth depressions (Rt) during the drought, ranged from ca. 20% at Horb to almost 40% at Heidelberg and Weithard. We also found significant models for Rc considering the elevation as negative predictor variable or testing the interaction between site and treatment. However, these models had poor AIC values (AIC-Delta between +24 and +73) and were therefore rejected (Table 7).

### Growth Resilience After Drought (Rs)

Also for the growth resilience after drought (Rs), site was the most important predictor in models for both drought intensities.

For moderate droughts, the model with best AIC (=−192) clearly indicated no treatment effect for Rs (*p* = 0.46). The only model with a significant predictor included the factor site but had already a slightly poorer AIC (Delta +6; see Table 8). The two sites with highest growth depression during and best recovery after moderate drought years, Hospital and Weithard, also had the highest resilience of radial growth (Rs) (>1.1). However, also the remaining sites showed Rs indices of at least 1.0 which indicated that there were no medium-term growth reductions following moderate droughts (Figure 3E).

**Table 8:** Model results (glm) for the BAI resilience (Rs) of *P. abies* after drought.

	Predictor for Rs							
Drought type	Treatm. control	Site	mai_100_	Stand age at drought	Elevation	AIC	Delta	AIC weight
**Moderate**	x					−192	0	0.75
(*N* = 270)			−0.0014			−188	4	0.13
				−0.0011		−186	6	0.06
		**X**				−186	6	0.05
	x		−0.0012			−181	11	0.00
**Severe**		**X**				−172	0	0.85
(*N* = 270)	**X (**−**5.8%)**	**X**				−169	3	0.15
	x	**X**		0.0012		−156	16	0.00
					**0.0004**	−153	19	0.00
	**X (**−**5.2%)**				**0.0004**	−149	23	0.00

*Only the best fitting five models according to the AIC values are listed for moderate droughts (SPI −1 to −1.49) and severe droughts (SPI −1.5 to −1.99), respectively. X indicates significant factorial predictors, x indicates non-significant factorial predictors and bold figures provide the slope of significant continuous predictors. For the factor “Treatment” also the relative intercept change from control to limed treatment is listed.*

For severe droughts, the two best models with AICs of −172 and −169 (Table 8) identified relatively wide, site-specific variations of Rs and a general (i.e., not site-specific) treatment effect which indicated a 6% better resilience of BAI in trees on limed sites compared to control trees. At Horb (Rs = 0.8), Weithard (Rs = 0.9), and Heidelberg (Rs = 0.9), the mean radial growth in the 5 years after severe droughts did not completely return to the pre-drought growth levels, in spite of the high recovery rates described above (Figure 3D,F). In contrast, all sites with high growth resistance to severe droughts (Bad Waldsee, Herzogenweiler, Hospital) also showed favorable Rs indices ≥ 1.0.

### Influence of Time Period After First Lime Application on Rc and Rs

To further study the observed treatment effect for recovery and resilience of radial growth after severe droughts, Δ*t* – the time period between first lime application and the drought year – was added to the best models described above. For Rc, neither a general nor a treatment-specific influence of Δ*t* could be detected. In contrast, a significantly positive effect of Δ*t* was found for the resilience of radial growth (Rs). However, this effect of Δ*t* could not be assigned to liming as there was no interaction between Δ*t* and treatment (Table 9).

**Table 9:** Model results (glm) testing the influence of time period after liming (Δ*t*) on BAI recovery (Rc) and resilience (Rt) of *P. abies* after severe drought (SPI −1.5 to −1.99).

	Predictor (severe droughts only)					
	Treatment	Site	Δt	Δ*t* × Treatment	AIC	Delta	AIC weight
**Rc Models**	**X**	**X**	0.0012		292	0	0.99
(*N* = 139)	**X**	**X**	0.0020	x	301	9	0.01
**Rs Models**	**X**	**X**	**0.0287**		221	0	0.97
(*N* = 139)	**X**	**X**	**0.0227**	x	228	7	0.03

*X indicates significant factorial predictors, x denotes non-significant factorial predictors, bold figures indicate the slope of a significant continuous predictor.*

## Discussion

This study investigated whether the drought tolerance of Norway spruce can be improved through repeated lime applications. Our results showed that there was no general increase in drought tolerance after liming. Differences in the radial growth response during and after drought years depended on drought intensity and were in most cases to a greater proportion explained by site than by liming. Furthermore, the timing of the identified drought events of different intensity in combination with the number and dosage of lime applications must be considered when interpreting our results.

### Growth Resistance (Rt)

There was no general treatment effect of liming on resistance of radial growth. In moderate droughts, the response of Rt to liming in Norway spruce was almost negligible; only 3% higher than in control trees. This weak response of Rt to liming corresponded with small growth changes in moderate drought years across all sites investigated.

In contrast, site-specific growth depressions in years classified as severe drought have been much more prominent (up to −40%). Nevertheless, we found also for severe droughts neither a general nor a site-specific improvement of Rt in trees of limed treatments. To our knowledge, there are no other studies on the effect of liming on the drought responses of “Llorets growth indices” (Rt, Rc, and Rs) that could be used for comparison of our findings. However, it was demonstrated previously that the drought resistance of radial growth in Norway spruce could also not be improved by thinning (Kohler et al., 2010; Sohn et al., 2012), which is a silvicultural option to improve stability and vitality of trees through enlarging their growing space. These studies showed that radial growth reduction in larger trees with presumably larger individual root systems (from heavy thinning) was as large as that of trees with less growing space. It demonstrated that the size of the root system does not confer any advantages, if the entire soil profile falls dry during drought years. We may assume that the same would apply to root systems that may be more intense as a result of liming. The advantage that liming could provide during drought years is therefore an increase in deep roots that would ensure that tree may access soil or ground water for longer periods. However, that was obviously not the case.

Severe droughts were dated between 1985 and 2003, only at Bad Waldsee we identified a severe drought in 2015 (Table 5). This means that our models for severe droughts mainly included data from the first 18 years of the experiment considering the short to mid-term effect of the first lime application in 1985 only (3 t dolomite ha^−1^) whereas the fine-root distributions shown in Figure 1 were assessed in 2015, and therefore influenced through the second, distinctly higher application rate of lime in 2003 (6 t dolomite ha^−1^).

Importantly, our results on the resistance of radial growth in severe drought years do not confirm findings of previous studies where an increase of weather-sensitivity of radial growth in limed trees was detected (van der Perre et al., 2012). This had been explained with the shallow root system of Norway spruce and the limited depth effect of a single lime application. Other studies had found even more shallow root systems of Norway spruce after liming, indicating an increased risk of drought stress through the treatment (Kreutzer, 1995; Helmisaari and Hallbäcken, 1999). However, based on results from our study, we can exclude an increased risk for drought stress after once-only liming in the mid- to long-term at all study sites. Unfortunately, we have no data on possible changes in fine-root distributions within the 18-year period after the first liming. Yet, it has already been shown that even after single lime applications, a deeper and more homogeneous root penetration as well as higher fine-root densities of Norway spruce can be achieved in the long-term (Schäffer, 2006). However, the lime dosage in that study was distinctly higher (10 t ha^−1^) than the commonly applied quantity of 3 t ha^−1^, which was also the dosage of the first liming in our study.

Within our 30-year observation period, we identified only two extreme droughts, namely in 2003 at site Bad Waldsee and in 2015 at site Horb. For the extreme drought at Bad Waldsee, we found also no treatment effect on the resistance of BAI suggesting that the assumptions discussed above already for severe droughts can be applied here, too. The second liming that occurred at this site in 2003, is unlikely to have had any direct impact on the growth reaction in that drought year as again slowly soluble dolomite rock powder was applied, even if the dosage of 6 t ha^−1^ was the double quantity of the first application in 1985.

In contrast, there was a strongly positive effect of liming on Rt (+20%) for the extreme drought 2015 at Horb. Several explanations are possible for this phenomenon. The root survey from 2015 showed increased fine-root densities at Horb after the two lime applications in 1985 and 2003, both in the upper mineral soil (0 to 5 cm depth) and at medium depth (20–25 cm) which may explain the higher resistance of limed trees at Horb in 2015, if the increased rooting density led to a later onset of drought stress (Figure 1).

Furthermore, the better Rt in limed trees could also have been influenced by the third lime application at Horb, where a mixture of dolomite rock powder and readily soluble wood ash was carried out in May 2015. Wood ash admixtures are considered to improve rapidly the supply of plant available potassium (K) (Wilpert et al., 2013). In previous studies, a distinct and prompt increase of foliar K concentrations in Norway spruce was detected in response to wood ash application within a short-term period (Schäffer, 2002; Wilpert, 2002). Foliar K content is assumed to play an important role in the drought stress tolerance of trees, since cellular K^+^ fluxes control the opening and closing of stomata (Kadereit and Strasburger, 2013). Unfortunately, foliar element analyses of limed and control trees in or after the drought 2015 are not available yet. However, potassium availability is limited in many forest soils in Baden-Württemberg owing to a selective potassium depletion on soil aggregates surfaces which has been observed especially on loamy sites (Hildebrand, 1990; Wilpert et al., 1993; Wilpert and Hildebrand, 1994). At Horb, the soil texture class was monitored as clay-loam (Table 1), so that limitations in the foliar potassium nutrition cannot be excluded there. Nevertheless, whether improved stomatal control may be responsible for such a strong increase in radial growth resistance is questionable.

The 35-year age difference between trees limed and the control stand could potentially also have influenced the different Rt response to extreme drought at Horb. However, this can be largely excluded as in none of the Rt models, regardless of drought intensity, the variable “stand age at drought” has been found to be a significant predictor (Table 6). Likewise, the predictor “site index” – which was a significant predictor of Rt when used in a mono-variable model – had no explanatory power at all in the Rt model considering the interaction between “site” and “treatment.” This indicated that the improved Rt was not a general site index effect but a site-specific treatment effect which has been developed after 30 years at Horb. Otherwise, the site index should have been a significant predictor of Rt already in previous droughts, which was not the case (Table 6).

### Recovery and Resilience of Growth (Rc and Rs)

The response of Rc and Rs to liming was different depending on the drought intensity. After moderate drought, we found no treatment effect on both response variables. All sites showed resilience indices of at least 1.0, which means that growth returned completely to its respective pre-drought level at all sites. This was not unexpected as it corresponded to the only little growth changes of Rt in moderate drought years so that the absence of a treatment effect also for Rc and Rs was not surprising.

After severe droughts, both Rc and Rs were found to be significantly higher in trees from limed treatments compared to control trees. This means that liming had an impact on Llorets-growth indices similar as found in previous studies for thinning: no effect on growth resistance, but significant better growth recovery of treated Norway spruces in post drought years. However, the effect size of heavy thinning was much stronger with a Rc improvement that was mostly higher than 25% (Kohler et al., 2010; Sohn et al., 2012). This higher recovery was only possible because trees in these thinning experiments that were carried out at other sites, both from thinned and unthinned plots, were more strongly affected during the drought (lower Rt values of less than 0.5) than in the liming experiments. In our study, the treatment effect of liming was only of moderate size with an Rc and Rs improvement of +8% and +6%, respectively. Otherwise, it must be seen that this positive response to liming was the result of once-only applications of dolomite rock powder in the common dosage of 3 t ha^−1^. Thus, our results provided evidence that already after a single lime application a measurable improvement of drought tolerance in Norway spruce can be achieved in the short- to medium-term. Since we have no data on fine-root distribution, sap flow or foliar element contents for this period, it is not possible to point to the underlying mechanisms.

### Influence of Time Span Between Liming and Drought (Δ*t*) on Rc and Rs

We could not find any influence of Δ*t* on the recovery or resilience of radial growth after severe droughts. For this, different explanations are possible. As already mentioned, severe droughts occurred predominantly in the 18-year period after the first lime application. Perhaps this time period was not long enough to detect any time effect of liming in our models, especially when considering that the observed effect of liming on Rc and Rs was only of moderate size.

Furthermore, increasing stand ages within the 18-year period could have blurred possible time effects of liming on Rc and Rs. However, we could not find any significant effect of the predictor “stand age at drought event” in our models. In the literature, contrasting results on the role of stand age on climate sensitivity of tree growth have been reported. No general effect of tree age on climate sensitivity of radial growth has been found in Zang et al. (2014) while in other studies both increased sensitivity (e.g., Martinez-Vilalta et al., 2012) or also decreased sensitivity with advancing tree age were described (Szeicz and MacDonald, 1995). Compared to these studies, the period of changing stand age was relatively short so that the absent “stand age effect” in our models was reasonable.

A liming-specific Δ*t* effect could also be obscured by the general recovery of soil chemical and forest nutritional conditions which has been reported for many forest sites in Germany in the second nation-wide forest soil survey (Wellbrock et al., 2016). This improvement of environmental conditions for forest growth was mainly caused by the reduction of the airborne deposition of sulfur which led also in Baden-Württemberg - where the sites of our study were located – to a measurable increase of pH-values in many forest soils (Hug et al., 2005). A general trend of increasing growth and vitality of Norway spruce since the mid of the 1980s has also been reported in Uhl et al. (2013).

## Conclusion

This is the first study that tested the influence of repeated lime applications on the growth response of Norway spruce trees to droughts of different intensity across a variety of sites in south-western Germany. We showed that liming did not generally reduce the impact of drought during the event. This would only be possible, if liming increased water holding capacity of the soil, improved the maximum rooting depth to increase the soil volume from which trees can extract water, or fostered physiological drought responses such as stomatal control to reduce transpiration. The latter two may have occurred to some extent at one site that was additionally treated with wood ash 30 years after the first lime application, leading to high concentrations of soluble potassium in soil.

Despite the lack in stress reduction during drought events, liming improved the drought tolerance of trees. In contrast to the assumptions in previous studies, our results indicated that even a single lime application can lead to moderate improvements of growth recovery and resilience after severe droughts. Even if this liming effect was only moderate, it may help to reduce the stress period in trees, which in turn may reduce the vulnerability to secondary, drought-related pests and pathogens. This is particularly relevant in case of Norway spruce.

However, the relatively small influence of liming on drought tolerance of Norway spruce will not facilitate the cultivation of this species at sites with high drought risk. Apart from the above mentioned increase in vulnerability to pest and pathogens, also distinctly higher risk of drought induced stem-crack damages must be expected, especially in younger and fast growing stands of Norway spruce on fertile soils (Kohler et al., 2017). Thus, Norway spruce dominated stands growing at sites with high drought risks should be converted into mixed stands with more stable and climate-change tolerant trees species (Spiecker, 2003; Vitali et al., 2018).

## Author Contributions

MK made substantial contributions to the conception and design of the study, analysis and interpretation of data, and is the lead author of the manuscript. JK was mainly responsible for data acquisition and has participated in data analysis and writing. JH made substantial contributions to data acquisition and interpretation of data. PH, LJ, and HP contributed to data acquisition, data interpretation and writing. JB had the idea for this study. KW and JB contributed to data interpretation and writing.

## Conflict of Interest Statement

The authors declare that the research was conducted in the absence of any commercial or financial relationships that could be construed as a potential conflict of interest. The handling Editor declared a shared affiliation, though no other collaboration, with several of the authors PH, LJ, HP, and KW at the time of review.

## Footnotes

1 www.r-project.org

## References

[B1] Albrecht A. Hanewinkel M. Bauhus J. Kohnle U. (2012). How does silviculture affect storm damage in forests of south-western Germany? results from empirical modeling based on long-term observations. *Eur. J. For. Res.* 131 229–247. 10.1007/s10342-010-0432-x

[B2] BMELV (2018). *National Forest Inventory (BWI).* Available at: http://www.bundeswaldinventur.de

[B3] Bunn A. G. (2008). A dendrochronology program library in R (dplR). *Dendrochronologia* 26 115–124. 10.1016/j.dendro.2008.01.002

[B4] Buras A. (2017). A comment on the expressed population signal. *Dendrochronologia* 44 130–132. 10.1016/j.dendro.2017.03.005

[B5] Campelo F. García-González I. Nabais C. (2012). detrendeR – A graphical user interface to process and visualize tree-ring data using R. *Dendrochronologia* 30 57–60. 10.1016/j.dendro.2011.01.010

[B6] Dietrich H. Wolf T. Kawohl T. Wehberg J. Kändler G. Mette T. (2018). Temporal and spatial high-resolution climate data from 1961-2100 for the German National Forest Inventory (NFI). *Ann. For. Sci.* 76:6 10.1007/s13595-018-0788-5

[B7] Fritts H. C. (1976). *Tree Rings and Climate.* London: Academic Press.

[B8] Fritts H. C. Shatz D. J. (1975). Selecting and chracterizing tree-ring chronologies for dendroclimatic analysis. *Tree Ring Bull.* 35 31–40.

[B9] Gutachterausschuss forstliche Analytik (2009). *Handbuch Forstliche Analytik.* Bonn: Bundesministerium für Verbraucherschutz, Ernährung und Landwirtschaft.

[B10] Hanewinkel M. Cullmann D. A. Schelhaas M.-J. Nabuurs G.-J. Zimmermann N. E. (2013). Climate change may cause severe loss in the economic value of European forest land. *Nat. Clim. Change* 3 203–207. 10.1038/nclimate1687

[B11] Hayes M. J. Svoboda M. D. Wilhite D. A. Vanyarkho O. V. (1999). Monitoring the 1996 drought using the standardized precipitation index. *Bull. Am. Meteorol. Soc.* 80 429–438. 10.1175/1520-0477(1999)080<0429:MTDUTS>2.0.CO;2

[B12] Helmisaari H. S. Hallbäcken L. (1999). Fine-root biomass and necromass in limed and fertilized Norway spruce (*Picea abies* (L.) Karst.) stands. *For. Ecol. Manag.* 119 99–110. 10.1016/S0378-1127(98)00514-3

[B13] Hildebrand E. E. (1990). Die bedeutung der bodenstruktur für die waldernährung, dargestellt am beispiel des kaliums. *Forstwissenschaftliches Centralblatt* 109 2–12. 10.1007/BF02741616

[B14] Huettl R. F. (1989). Liming and fertilization as mitigation tools in declining forest ecosystems. *Water Air Soil Pollut.* 44 93–118. 10.1007/BF00228781

[B15] Hug R. Hepp R. Wilpert K. V. (2005). *18 Jahre Depositionsmessnetz der Forstlichen Versuchs- und Forschungsanstalt Baden-Württemberg.* Freiburg im Breisgau: Berichte Freiburger Forstlicher Forschung Heft.

[B16] Kadereit J. W. Strasburger E. (2013). *Strasburger’s Plant Sciences. Heidelberg New York Dordrecht.* London: Springer.

[B17] Kohler M. Kiehne J. Borchers J. Bauhus J. (2017). How do h/d ratio and site index affect the occurrence of stem cracks in young Norway spruce (*Picea abies* L. Karst) stands? *Allg. Forst- u. Jagdzeitschrift* 188 197–209.

[B18] Kohler M. Sohn J. Nägele G. Bauhus J. (2010). Can drought tolerance of Norway spruce (*Picea abies* (L.) Karst.) be increased through thinning? *Eur. J. For. Res.* 129 1109–1118. 10.1007/s10342-010-0397-9

[B19] Kreutzer K. (1995). Effects of forest liming on soil processes. *Plant Soil* 168 447–470. 10.1007/BF00029358

[B20] Kunz J. Löffler G. Bauhus J. (2018). Minor European broadleaved tree species are more drought-tolerant than Fagus sylvatica but not more tolerant than *Quercus petraea*. *For. Ecol. Manag.* 414 15–27. 10.1016/j.foreco.2018.02.016

[B21] Landesanstalt für Umwelt Messungen und Naturschutz Baden-Württemberg [LUBW] (2013). *Zukünftige Klimaentwicklung in Baden-Württemberg: Perspektiven aus regionalen Klimamodellen - Kurzfassung.* Karlsruhe: LUBW.

[B22] LeBlanc D. C. (1990). Relationships between breast-height and whole-stem growth indices for red spruce on Whiteface Mountain. New York. *Can. J. For. Res.* 20 1399–1407. 10.1139/x90-185

[B23] Lebourgeois F. Bréda N. Ulrich E. Granier A. (2005). Climate-tree-growth relationships of European beech (*Fagus sylvatica* L.) in the French permanent plot network (RENECOFOR). *Trees* 19 385–401. 10.1007/s00468-004-0397-9

[B24] Lloret F. Keeling E. G. Sala A. (2011). Components of tree resilience: effects of successive low-growth episodes in old ponderosa pine forests. *Oikos* 120 1909–1920. 10.1111/j.1600-0706.2011.19372.x

[B25] Martinez-Vilalta J. Lopez B. C. Loepfe L. Lloret F. (2012). Stand- and tree-level determinants of the drought response of Scots pine radial growth. *Oecologia* 168 877–888. 10.1007/s00442-011-2132-8 21983639

[B26] Mayer H. Holst T. Brugger U. Kirchgässner A. (2005). Trends der forstlich relevanten klimavariablen lufttemperatur und niederschlag im südwesten deutschlands von 1950 bis 2000. *Allgemeine Forst und Jagdzeitung* 176 45–56.

[B27] McKee T. B. Doesken N. J. Kleist J. (1993). “The relationship of drought frequency and duration to time scales,” in *Proceedings of the 8th Conference on Applied Climatology*, (Boston: American Meteorological Society), 179–184.

[B28] Neves J. (2012). *Package ‘spi’. Compute SPI index.* Wien: CRAN.

[B29] Peterson G. Allen C. R. Holling C. S. (1998). Ecological resilience, biodiversity, and scale. *Ecosystems* 1 6–18. 10.1007/s100219900002

[B30] Pimm S. L. (1984). The complexity and stability of ecosystems. *Nature* 317 321–326. 10.1038/307321a0

[B31] Ponette Q. Dufey J. E. Weissen F. (1997). Downward movements of dolomite, kieserite or a mixture of CaCO3 and kieserite through the upper layers of an acid forest soil. *Water Air Soil Pollut.* 95 353–379. 10.1007/BF02406174

[B32] Quiring S. M. (2009). Developing objective operational definitions for monitoring drought. *J. Appl. Meteor. Climatol.* 48 1217–1229. 10.1175/2009JAMC2088.1

[B33] Schaaf W. Hüttl R. F. (2006). Experiences with liming in European countries – results of long-term experiments. *J. For. Sci.* 52 35–44. 10.17221/10158-JFS

[B34] Schäffer J. (2002). meliorationswirkung und ökosystemare risiken von holzascheausbringung auf waldböden südwestdeutschlands. *Berichte Freiburger Forstliche Forschung Heft* 43 39–52.

[B35] Schäffer J. (2006). Brauchen wir ein langfristiges Kalkungskonzept? *FVAeinblick* 2 7–10.

[B36] Schober R. (1995). *Ertragstafeln wichtiger Baumarten bei verschiedener Durchforstung.* Frankfurt am Main: J. D. Sauerländers Verlag.

[B37] Schweingruber F. H. (1983). *Der Jahrring: Standort, Methodik, Zeit und Klima in der Dendrochronologie.* Bern: Haupt.

[B38] Sohn J. A. Kohler M. Gessler A. Bauhus J. (2012). Interactions of thinning and stem height on the drought response of radial stem growth and isotopic composition of Norway spruce (*Picea abies*). *Tree Physiol.* 32 1199–1213. 10.1093/treephys/tps077 22961177

[B39] Spiecker H. (1991). Liming, nitrogen and phosphorus fertilization and the annual volume increment of Norway spruce stands on long-term permanent plots in Southwestern Germany. *Fertilizer Res.* 27 87–93. 10.1007/BF01048611

[B40] Spiecker H. (2003). Silvicultural management in maintaining biodiversity and resistance of forests in Europe—temperate zone. *J. Environ. Manage.* 67 55–65. 10.1016/S0301-4797(02)00188-3 12659804

[B41] Szeicz J. MacDonald G. (1995). Dendroclimatic reconstruction of summer temperatures in northwestern Canada since A. D. 1638 based on age-dependent modeling. *Quat. Res.* 44 257–266. 10.1006/qres.1995.1070

[B42] Uhl E. Ammer C. Spellmann H. Schölch M. Pretzsch H. (2013). Zuwachstrend und Stressresilienz von Tanne und Fichte im Vergleich. *Allgemeine Forst und Jagdzeitung* 184 278–292.

[B43] Ulrich B. (1986). Die Rolle der Bodenversauerung beim Waldsterben: Langfristige Konsequenzen und forstliche Möglichkeiten. *Forstwissenschaftliches Centralblatt* 105 421–435. 10.1007/BF02741750

[B44] van der Maaten-Theunissen M. van der Maaten E. Bouriaud O. (2015). pointRes: an R package to analyze pointer years and components of resilience. *Dendrochronologia* 35 34–38. 10.1016/j.dendro.2015.05.006

[B45] van der Perre R. Jonard M. André F. Nys C. Legout A. Ponette Q. (2012). Liming effect on radial growth depends on time since application and on climate in Norway spruce stands. *F. Ecol. Manag.* 281 59–67. 10.1016/j.foreco.2012.06.039

[B46] Vitali V. Büntgen U. Bauhus J. (2017). Silver fir and Douglas fir are more tolerant to extreme droughts than Norway spruce in south-western Germany. *Glob. Change Biol.* 23 5108–5119. 10.1111/gcb.13774 28556403

[B47] Vitali V. Büntgen U. Bauhus J. (2018). Seasonality matters—the effects of past and projected seasonal climate change on growth of native and exotic conifers in Central Europe. *Dendrochronologia* 48 1–9. 10.1016/j.dendro.2018.01.001

[B48] Wellbrock N. Bolte A. Flessa H. (2016). *Dynamik und räumliche Muster forstlicher Standorte in Deutschland - Ergebnisse der Bodenzustandserhebung im Wald 2006 bis 2008: Thünen Report 43.* Braunschweig: Johann Heinrich von Thünen-Institut.

[B49] West P. W. (1980). Use of diameter increment and basal area increment in tree growth studies. *Can. J. For. Res.* 10 72–77. 10.1139/x80-012

[B50] Wigley T. Briffa K. R. Jones P. D. (1984). On the average value of correlated time series, with applications in dendroclimatology and hydrometeorology. *J. Clim. Appl. Meteorol.* 23 201–213. 10.1175/1520-0450(1984)023<0201:OTAVOC>2.0.CO;2

[B51] Wilpert K. V. (2002). Eckpunkte und wissenschaftliche Begründung eines Holzasche-Kreislaufkonzepts. *Berichte Freiburger Forstlicher Forschung Heft* 43 17–28.

[B52] Wilpert K. V. Hartmann P. Schaeffer J. (2013). *Regenerationsorientierte Bodenschutzkalkung: Merkblatt 54.* Freiburg im Breisgau: FVA Freiburg.

[B53] Wilpert K. V. Hartmann P. Schäffer J. (2016). Quality control in a wood ash re-cycling concept for forests. *VGB PowerTech* 96 67–72.

[B54] Wilpert K. V. Hildebrand E. E. (1994). Stoffeintrag und Waldernährung in Fichtenbeständen Baden-Württembergs. *Forst und Holz* 49 629–632. 25626619

[B55] Wilpert K. V. Hildebrand E. E. Huth T. (1993). *Ergebnisse des Praxis-Großdüngeversuches - Abschlußbericht über die Anfangsaufnahmen (1985/86) und die Endaufnahmen (1989/90): Mitteilungen der FVA Baden-Württemberg Heft 171.* Freiburg: FVA, 133S.

[B56] Zang C. Hartl-Meier C. Dittmar C. Rothe A. Menzel A. (2014). Patterns of drought tolerance in major European temperate forest trees: climatic drivers and levels of variability. *Glob. Change Biol.* 20 3767–3779. 10.1111/gcb.12637 24838398

